# HDAC6 inhibitor WT161 downregulates growth factor receptors in breast cancer

**DOI:** 10.18632/oncotarget.19019

**Published:** 2017-07-05

**Authors:** Teru Hideshima, Ralph Mazitschek, Jun Qi, Naoya Mimura, Jen-Chieh Tseng, Andrew L. Kung, James E. Bradner, Kenneth C. Anderson

**Affiliations:** ^1^ Department of Medical Oncology, Dana-Farber Cancer Institute and Harvard Medical School, Boston, MA, USA; ^2^ Center for Systems Biology, Massachusetts General Hospital, Harvard Medical School, Boston, MA, USA; ^3^ Lurie Family Imaging Center, Dana-Farber Cancer Institute, Boston, MA, USA; ^4^ Department of Pediatric Oncology, Dana-Farber Cancer Institute and Children's Hospital Boston, Boston, MA, USA; ^5^ Department of Hematology, Chiba University Hospital, Chiba, Japan; ^6^ PerkinElmer Inc., Hopkinton, MA, USA; ^7^ Memorial Sloan Kettering Cancer Center, New York, NY, USA; ^8^ Novartis Institutes for BioMedical Research, Cambridge, MA, USA

**Keywords:** histone deacetylase inhibitor, breast cancer, estrogen receptor, epidermal growth factor receptor, proteasome inhibitor

## Abstract

We have shown that WT-161, a histone deacetylase 6 (HDAC6) inhibitor, shows remarkable anti-tumor activity in multiple myeloma (MM) in preclinical models. However, its activity in other type of cancers has not yet been shown. In this study, we further evaluated the biologic sequelae of WT161 in breast cancer cell lines. WT161 triggers apoptotic cell death in MCF7, T47D, BT474, and MDA-MB231 cells, associated with decreased expression of EGFR, HER2, and ERα and downstream signaling. However, HDAC6 knockdown shows that cytotoxicity and destabilization of these receptors triggered by WT161 are not dependent on HDAC6 inhibition. Moreover WT161 analog MAZ1793, which lacks HDAC inhibitory effect, similarly triggers cell line growth inhibition and downregulation of these receptors. We also confirm that WT161 significantly inhibits in vivo MCF7 cell growth, associated with downregulation of ERα, in a murine xenograft model. Finally, WT161 synergistically enhances bortezomib-induced cytotoxicity, even in bortezomib-resistant breast cancer cells. Our results therefore provide the rationale to develop a novel class of therapeutic agents targeting growth pathways central to the pathogenesis of breast cancer.

## INTRODUCTION

Histone proteins are localized in the nuclei of all eukaryotic cells and, as the predominant protein components of chromatin, play a major role in modulating the binding of transcription factors to DNA. The accessibility of expressed genes and the assembly of transcriptional complexes are influenced by post-translational side-chain acetylation (Kac) of lysine residues on unstructured amino-terminal histone tails. Acetylation of lysine is regulated by the balance of activities of two key classes of enzymes: histone acetyltransferases (HAT) and histone deacetylases (HDAC). Recent studies have shown that HDAC inhibitors are promising anti-tumor agents for various malignancies; consequently, structurally diverse HDAC inhibitors have either been purified from natural sources or developed through synthetic efforts as chemical probes and therapeutic agents.

HDAC6 is a class IIB lysine deacetylase which exerts various biologic activities in different cell types [1]. In multiple myeloma (MM), we have shown that HDAC6 knockdown or treatment with the selective small molecule HDAC6 inhibitors tubacin [2], ricolinostat [3], and WT161 [4], synergistically enhances bortezomib-induced cytotoxicity. In breast cancer cells, HDAC6 is a critical component of the invasive apparatus of tumor cells [5], and impacts epithelial organization of HER2-positive breast cancer cells [6]. HDAC6 also deacetylates HMGN2 to regulate STAT5a activity and breast cancer growth [7]. In clinical trials, levels of HDAC6 mRNA expression are a prognostic factor and marker of endocrine responsiveness [8]. Specifically, patients with HDAC6-positive breast cancer have longer progression-free survival and have increased overall survival after tamoxifen treatment, compared to patients with HDAC6-negative tumors [9]. To date, however, the biologic significance of HDAC6 in breast cancer has not been fully elucidated.

In this study, we examined the role of HDAC6 in breast cancer pathogenesis using HDAC6 knockdown and HDAC6-selective inhibitor WT161 [4], in breast cancer cell lines. Since proliferation of breast cancer cells is mediated by transmembrane growth factor receptors and intracellular hormone/steroid receptors including epidermal growth factor receptor (EGFR), human epidermal growth factor receptor 2 (HER2), HER3, KIT, estrogen receptor (ER), and progesterone receptor (PGR), we examined the effects of WT161 on receptor mRNA and protein expression. WT161 downregulated EGFR, HER2, and ER on breast cancer cells, associated with significant tumor growth inhibition both *in vitro* and *in vivo* in a xenograft mouse model. Surprisingly, knock-down of HDAC6 did not demonstrate comparable biological effects, suggesting that the anti-proliferative activity of WT161 in breast cancer is not HDAC6 dependent. To explore this further, we prepared a synthetic analogue of WT161, MAZ1793, which lacks HDAC inhibitory activity, and observed comparable downregulation of growth receptors associated with a robust anti-proliferative response. In MM, we have demonstrated remarkable anti-proliferative activity of the proteasome inhibitor bortezomib in preclinical models [10, 11] and in patients with relapsed-refractory disease [12, 13], leading to its FDA approval. In contrast, the single agent activity of bortezomib in solid tumors including breast cancer is limited. Here we show that WT161 significantly enhances bortezomib-induced cytotoxicity, even in bortezomib-resistant breast cancer cells. Taken together, our data demonstrate that both WT161 and MAZ1793 trigger downregulation of growth factor receptors and growth inhibition in breast cancer cells, providing the preclinical rationale for the development and evaluation of derivatives for clinical evaluation to improve patient outcome in breast cancer.

## RESULTS

### WT161 induces cytotoxicity in breast cancer cell lines

**Figure 1:**
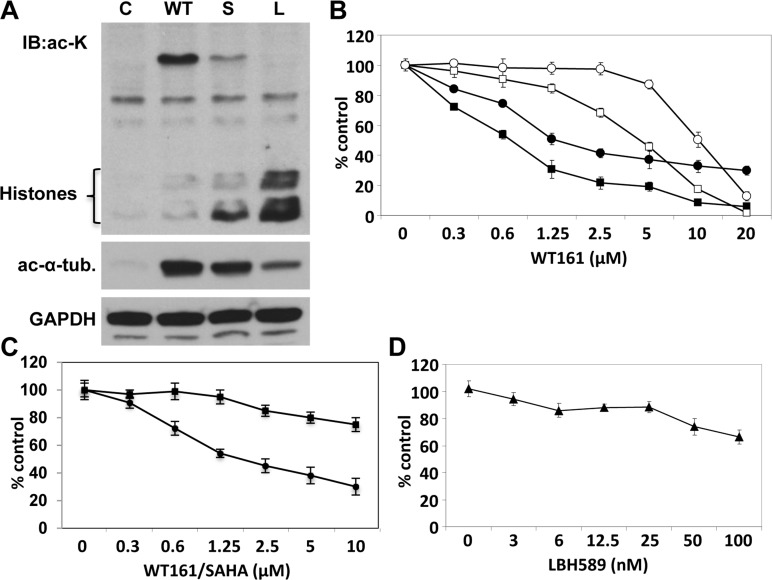
WT161 triggers cytotoxicity in breast cancer cell lines **A.** MCF7 cells were cultured in the presence of WT161 (WT, 1 μM), SAHA (S, 1 μM), or LBH589 (L, 50 nM) for 8h. Whole cell lysates were subjected to immunoblotting using anti-ac-lysine (polyclonal) Ab. **B.** MCF7 (●), T47D (■), BT474 (□), and MDA-MB231 (○) cells were cultured with WT161 (0 - 20 μM) for 72h. **C.** MCF7 cells were cultured with WT161 (●) or SAHA (■) (0 - 10 uM) for 72h. **D.** MCF7 cells were cultured with LBH589 (▲) (0 - 100 nM) for 72h. Percent survival relative to control was assessed by 72h MTT assay.

WT161 was synthesized as a novel selective HDAC6 inhibitor (Supplementary Figure 1). Since we have previously shown that HDAC6 inhibitor tubacin selectively induces acetylation of α-tubulin [14], we first examined protein acetylation in MCF7 cells induced by WT161, vorinostat (SAHA), and panobinostat (LBH589) using anti-ac-lysine and anti-ac-α-tubulin Abs. WT161 strongly increases acetylation of α-tubulin, without hyperacetylation of histones. In contrast, SAHA and LBH589 trigger acetylation of histones, with modest acetylation of α-tubulin. Since K40-Ac α-tubulin is a substrate of HDAC6, this result indicates that WT161 is a more selective inhibitor of HDAC6 than these pharmaceutical and investigational agents (Figure 1A). We next examined the growth inhibitory effect of WT161 in 4 breast cancer cell lines (MCF7, T47D, BT474, MDA-MB231). WT161 triggers significant growth inhibition in a dose-dependent fashion. Interestingly, MDA-MB231, a triple-negative cell line, shows the lowest sensitivity to WT161 (Figure 1B). To compare the growth inhibitory effect of WT161 with other HDAC inhibitors, we cultured MCF7 cells in the presence of SAHA (Figure 1C) and LBH589 (Figure 1D), which have shown potent cytotoxicity against MM cells at low μM and low nM range, respectively [15, 16]. MCF7 cells are relatively resistant to these non-selective HDAC inhibitors, but are sensitive to WT161.

### WT161 induces caspase cleavage and apoptosis, associated with XIAP downregulation

**Figure 2:**
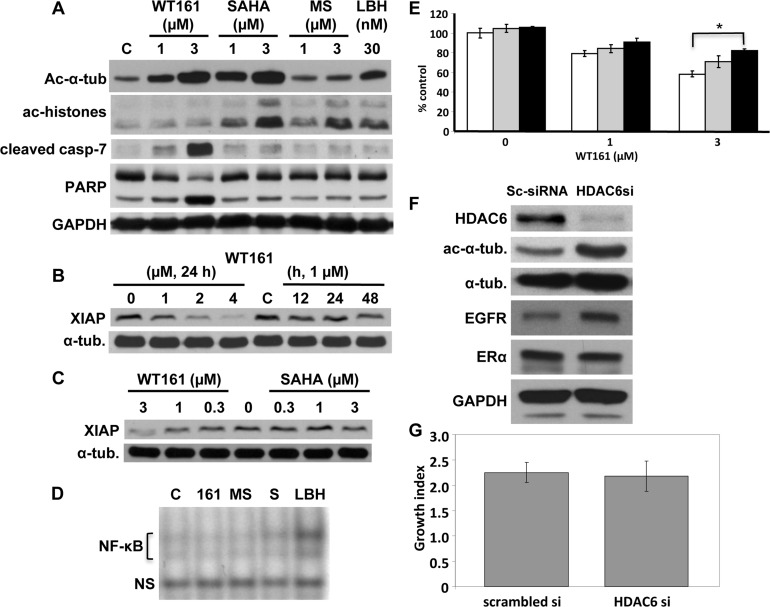
HDAC6 inhibition does not solely account for WT161-induced cytotoxicity **A.** MCF7 cells were cultured with WT161 (1 and 3 μM), SAHA (1 and 3 μM), MS275 (1 and 3 μM) or LBH589 (30 nM) for 24h. **B.** MCF7 cells were cultured with WT161 (1 - 4 μM) for 24h or with 1 μM WT161 for 0h - 48h. **C.** MCF7 cells were cultured with WT161 (0.3 - 3 μM) or SAHA (0.3 - 3 μM) for 24h. Whole cell lysates were immunoblotted with indicated Abs. **D.** MCF7 cells were treated with WT161 (161, 2 μM), MS275 (MS, 2 μM), SAHA (S, 2 μM) or LBH589 (LBH, 30 nM) for 24h. Nuclear extracts were subjected to EMSA. **E.** MCF7 cells were cultured for 48h with WT161 (1 and 3 μM), in the absence (□) or presence of 25 μM (■) and 50 μM (■) Z-VAD-FMK. Cell growth was assessed by MTT assay. **F.** MCF7 cells were transfected with scrambled or HDAC6 siRNA. After 48h culture, whole cell lysates were subjected to immunoblotting using indicated Abs. **G.** Scrambled- or HDAC6 siRNA-transfected MCF7 cells were cultured for 72h, and cell growth was assessed by MTT assay. *; *p* < 0.01.

We next examined mechanisms of action of WT161-induced cytotoxicity in MCF7 cells, a caspase-3 deficient cell line. Consistent with cytotoxicity profiling, WT161 strongly triggers caspase-7 and PARP cleavage in a dose-dependent fashion; in contrast, no caspase-7 or PARP cleavage is induced by SAHA, MS275, or LBH589 treatment (Figure 2A). A previous study has shown that X-linked inhibitor of apoptosis protein (XIAP) mediates anti-apoptosis in breast cancer cells [17]. We show that WT161 also downregulates XIAP expression in a dose- and time-dependent fashion (Figure 2B), whereas SAHA does not. (Figure 2C). Since a previous report shows that XIAP is a target molecule of NF-κB [18], we next examined whether WT161 inhibited NF-κB activity. Neither WT161 nor other HDAC inhibitors targeted NF-κB (Figure 2D). To confirm the role of caspase activation in WT161-induced cytotoxicity, we cultured MCF7 cells with WT161 in the presence of pan-caspase inhibitor Z-VAD-FMK. Cytotoxicity is significantly blocked by Z-VAD-FMK (Figure 2E), indicating that WT161-induced cytotoxicity is, at least in part, mediated *via* caspase-dependent apoptosis.

### HDAC6 inhibition does not solely account for WT161-induced cytotoxicity

To examine the significance of HDAC6 inhibition in breast cancer cell growth, we transiently transfected HDAC6 siRNA into MCF7 cells. HDAC6 expression was almost completely downregulated by HDAC6 siRNA, associated with upregulation of acetylated tubulin (Figure 2F). Surprisingly, no growth inhibitory effect was observed in HDAC6 siRNA transfected MCF7 cells (Figure 2G). These results strongly suggest that HDAC6 inhibition does not account for WT161-induced cytotoxicity.

### WT161 downregulates EGFR and ERα expression

**Figure 3:**
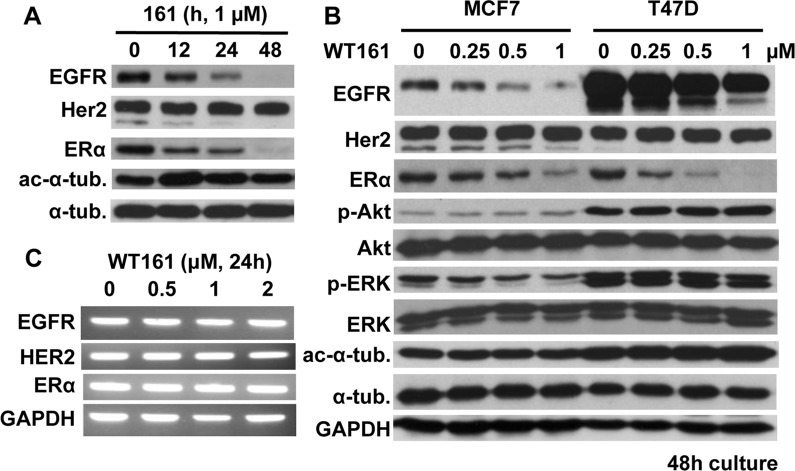
WT161 downregulates EGFR and ERα expression **A.** MCF7 cells were cultured with WT161 (1 μM) for 0 - 48h. **B.** MCF7 and T47D cells were cultured with WT161 (0 - 1 μM) for 48h. Whole cell lysates were subjected to immunoblotting with indicated Abs. **C.** MCF7 cells were cultured with WT161 (0 - 2 μM) for 24h. Total RNAs were extracted and subjected to PCR.

Previous studies have shown that loss of HDAC6 downregulates EGFR expression in lung cancer cells [19, 20]. We therefore next examined whether WT161 could downregulate EGFR and/or other receptor expression in breast cancer cells. WT161 decreases expression of EGFR and ERα in MCF7 cells in a time-dependent fashion (Figure 3A), without affecting the expression of estrogen-related receptor (ERR)-α (data not shown). WT161 dose-dependent downregulation of EGFR and ERα is also observed in T47D cells. Phospho-ERK, downstream of EGF/EGFR, is downregulated by WT161 treatment, but phospho-Akt/Akt is not altered (Figure 3B). RT-PCR shows that mRNA levels of EGFR, HER2, or ERα in MCF7 cells are not altered by WT161 treatment (Figure 3C). Importantly, we do not observe downregulation of EGFR or ERα in HDAC6 siRNA transfectants (data not shown). Taken together, these results suggest that WT161-triggered downregulation of EGFR and ERα in breast cancer cells is due to a post-transcriptional and HDAC6-independent event.

### HDAC inhibitors differentially downregulate receptor expression

**Figure 4:**
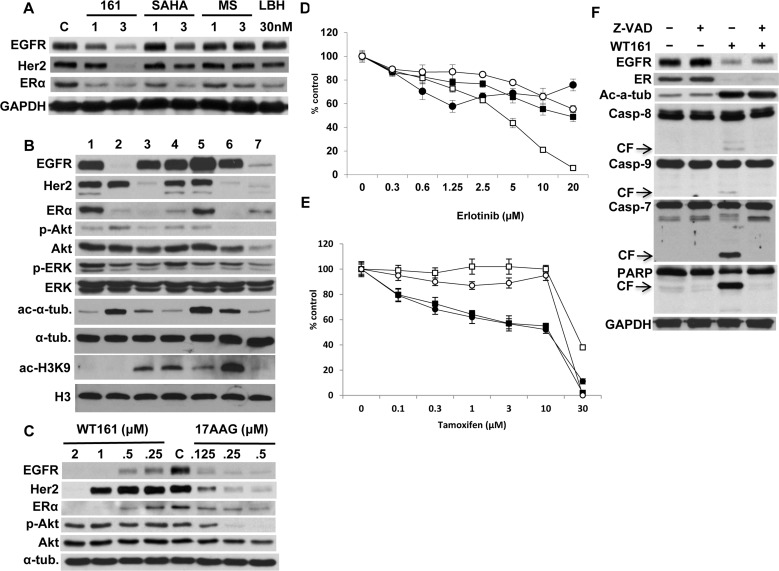
HDAC inhibitors differentially downregulate receptor expression **A.** MCF7 cells were cultured with WT161 (1 and 3 μM), SAHA (1 and 3 μM), MS275 (1 and 3 μM) or LBH589 (30 nM) for 24h. **B.** MCF7 cells were cultured with HDAC inhibitors for 48h. Lanes indicate; 1, DMSO control; 2, WT161 (1 μM); 3, trichostatin A (TSA, 0.5 μM); 4, MS275 (2.5 μM); 5, SAHA (1 μM); 6, LBH589 (50 nM); 7, 17AAG (0.5 μM). **C.** MCF7 cells were cultured with WT161 (0.25 - 2 μM) or 17AAG (0.125 - 0.5 μM) for 48h. Whole cell lysates were subjected to immunoblotting with indicated Abs. MCF7 (●), T47D (■), BT474 (□), and MDA-MB231 (○) cells were cultured with erlotinib **D.** or tamoxifen **E.** for 72h, and cell growth was assessed by MTT assay. **F.** MCF7 cells were cultured with WT161 (2 μM), in the absence or presence of Z-VAD-FMK (50 μM) for 24h. Whole cell lysates were subjected to immunoblotting with indicated Abs.

We next examined whether downregulation of EGFR and ERα was triggered by inhibition of other HDACs, in particular class I HDACs. MCF7 cells were treated with WT161, SAHA, MS275, and LBH589. WT161 (3 μM) markedly downregulates EGFR, HER2, and ERα at 24h; however, other HDAC inhibitors downregulate only ERα (Figure 4A). Previous studies have shown that HDAC6 inhibition/knockdown hyperacetylates Hsp90 and reduces its chaperone function [21, 22]. Since these receptors are known Hsp90 client proteins, we hypothesized that receptor downregulation might be due to blockade of Hsp90 *via* HDAC6 inhibition. We therefore next compared the patterns of receptor downregulation triggered by HDAC inhibitors *versus* Hsp90 inhibitor 17AAG in MCF7 cells. As expected, 17AAG (0.5 μM) significantly destabilizes EGFR, HER2, and ERα, as well as p-ERK and p-Akt. However, no common pattern of receptor downregulation is induced by HDAC inhibitors. For example, WT161 (1 μM) almost completely downregulates EGFR and ERα, whereas TSA (0.5 μM) downregulates HER2 and ERα. Importantly, WT161 does not inhibit, but rather increases p-Akt. These results are not correlated with ac-α-tubulin or ac-histone H3K9 (Figure 4B), suggesting that downregulation of receptors is unlikely to be caused by modulation of HDAC or Hsp90 function. We further performed a side-by-side comparison of WT161 *versus* 17AAG in 48h cultures of MCF7 cells. 17AAG (0.5 μM) significantly downregulates EGFR, HER2, ERα and p-Akt. Importantly, WT161 (2 μM) even more potently abrogated EGFR, HER2, and ERα expression than 17AAG; however, it does not alter p-Akt (Figure 4C). These results further support the view that WT161-induced downregulation of these receptors is independent of Hsp90. Of note, WT161 in combination with 17AAG markedly downregulates EGFR, HER2, and ERα, associated with enhanced cytotoxicity in MCF7 cells (Supplementary Figure 2).

To examine the significance of downregulation of receptor expression on breast cancer cell proliferation, MCF7, T47D, BT474, and MDA-MB231 cells were cultured with EGFR tyrosine kinase inhibitor erlotinib (Figure 4D) or ER antagonist tamoxifen (Figure 4E). Importantly, neither of these single agents triggered a growth inhibitory effect as pronounced as WT161. Similar results were observed using the potent ERα downregulator fulvestrant (Supplementary Figure 3). These results suggest that WT161- induced growth inhibition in breast cancer cells is not due to single receptor inhibition.

Recent studies have shown that CUDC-101, a hybrid small molecule inhibitor targeting EGFR/HER2 and HDACs, shows significant anti-tumor effects in various types of cancer cells [23]. We therefore compared the *in vitro* activity of WT161 *versus* CUDC-101 in breast cancer cells. WT161, in a dose dependent fashion, downregulates EGFR, HER2 and ERα; however, CUDC-101 (3 μM) shows only modest downregulation of EGFR and ERα, similar to that observed in cells treated with other HDAC inhibitors (Supplementary Figure 4A). Of note, CUDC-101 abrogates EGF-induced phosphorylation (Tyr1068) of EGFR in a dose-dependent fashion, associated with growth inhibition in breast cancer cells; however, WT161 had no effect on p-EGFR (Supplementary Figures 4B and 4C).

### WT161-triggered downregulation of EGFR or ERα is independent of caspase activation

Previous studies have shown that the C-terminus domain of EGFR is a target of caspases and subject to degradation during apoptosis [24]. Since WT161 induces caspase/PARP cleavage followed by apoptosis, we next examined whether downregulation of EGFR was due to caspase activation. WT161 significantly triggers caspase/PARP cleavage, which is completely abrogated by Z-VAD-FMK. However, WT161-induced downregulation of EGFR and ERα are not altered in the presence of Z-VAD-FMK, indicating that EGFR and ER are not substrates of caspases (Figure 4F).

### WT161 inhibits MCF-7 tumor growth in a mouse xenograft model

**Figure 5:**
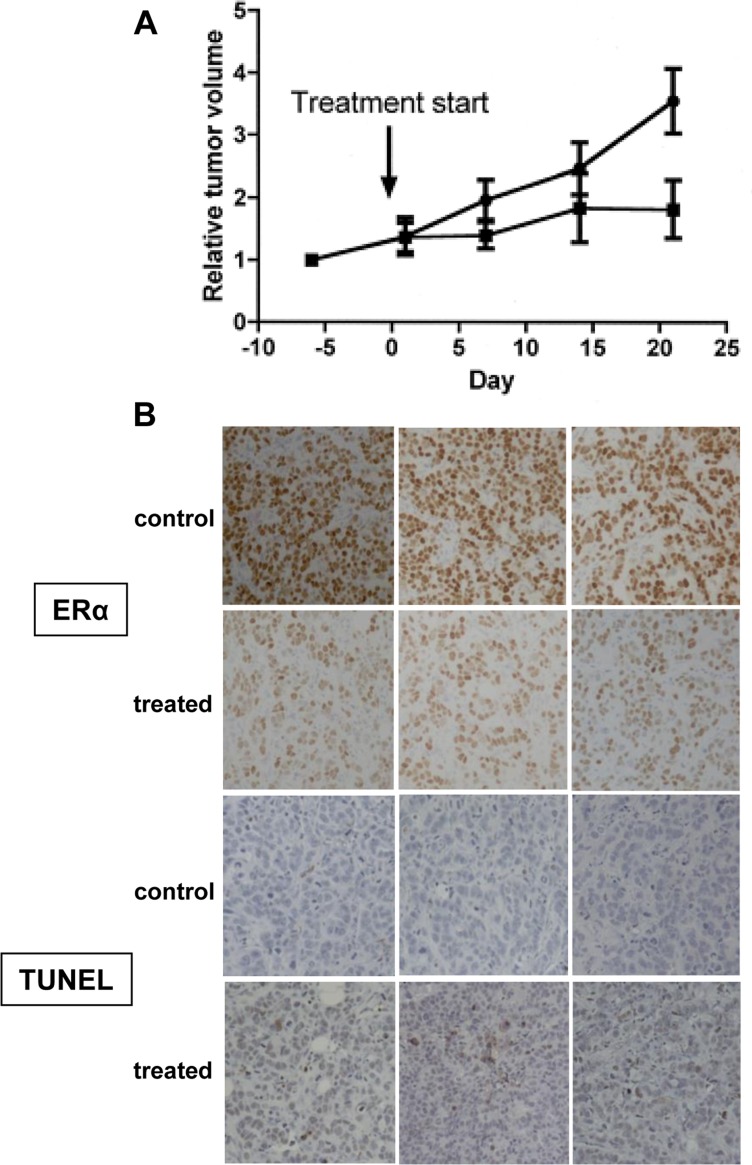
Anti-tumor efficacy of WT161 in MCF7 xenograft tumors **A.** Mice with established MCF7 xenograft tumors were assigned into cohorts receiving either daily intraperitoneal WT161 80 mg/kg (*n* = 7, ■) or vehicle control (*n* = 7,●) and relative tumor volume was calculated over time. **B.** After 3-week treatment, tumors harvested from mice were subjected to IHC analysis. Representative results (ER and TUNEL) from each cohort (control and WT161-treated) are shown.

We next examined the anti-tumor effect of WT161 in an MCF-7 mouse xenograft model. Mice with established tumors were treated with either vehicle control or WT161 at 80 mg/kg/day, i.p. for 3 weeks. As shown in Figure 5A, WT161 significantly inhibits the growth of MCF-7 xenografts (*p* = 0.035). Since ERα is the most sensitive to WT161 treatment and MCF-7 cells are responsive to ERα inhibitors (tamoxifen and fulvestrant), we performed immunohistochemical analyses for ERα expression and TUNEL staining on tumors excised from control and WT161-treated animals. Consistent with the findings above, ERα is markedly decreased in tumors treated with WT161 (Figure 5B). WT161-treated tumors also have increased TUNEL staining (Figure 5B), indicating increased tumor cell death.

### WT161 enhances bortezomib-induced cytotoxicity

**Figure 6:**
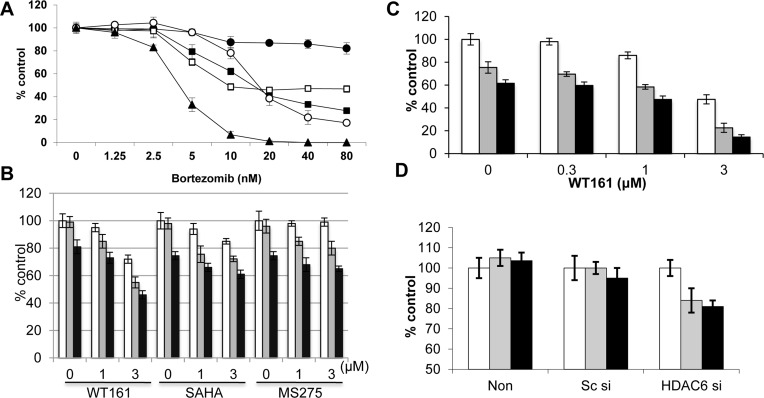
WT161 enhances bortezomib-induced cytotoxicity **A.** MCF7 (●), T47D (■), BT474 (□), MDA-MB231 (○), and RPMI8226 (▲) cells were cultured with bortezomib (1.25 - 80 nM) for 48h. **B.** MCF7 cells were cultured with WT161, SAHA, or MS275 (0 - 3 μM) in the presence of DMSO control (□), 10 nM (■) or 20 nM (■) bortezomib for 48h. **C.** BT474 cells were cultured for 48h with WT161 (0.3 - 3 μM) in the presence of DMSO control (□), and 5 nM (■) or 10 nM (■) bortezomib. **D.** MCF7 cells were transfected with scrambled siRNA (Sc si) or HDAC6 siRNA (HDAC6 si). Cells were then cultured in the presence of DMSO control (□), and 10 nM (□) or 20 nM (■) bortezomib for 48h. In all cases, cell growth was assessed by MTT assay and compared with control.

Bortezomib demonstrates remarkable clinical activity in MM; however, its activity as a single agent in breast cancer is limited. Moreover, compared to MM cell line RPMI8226, breast cancer cells are relatively resistant to bortezomib treatment. Specifically, MCF7 cells are resistant to treatment with bortezomib (Figure 6A) and other proteasome inhibitors including MG132 (Supplementary Figure 5) and lactacystin (data not shown). Since we have shown that HDAC6 selective inhibitor tubacin significantly enhances bortezomib-induced cytotoxicity in MM cell lines and patient MM cells [14], we examined whether WT161 or other HDAC inhibitors similarly enhanced anti-tumor activity of bortezomib in breast cancer cells. WT161 synergistically (CI = 0.85) enhances bortezomib-induced MCF7 cytotoxicity, whereas SAHA and MS275 show only a modest additive cytotoxic effect (Figure 6B). Similar results with WT161 are observed in BT474 cells (Figure 6C).

Since the growth inhibitory effect of WT161 as a single agent is independent of HDAC6, we next examined whether HDAC6 knockdown enhanced bortezomib-induced cytotoxicity. Interestingly, knockdown of HDAC6 triggers only modest increases in bortezomib-induced cytotoxicity in MCF7 cells (Figure 6D). We therefore further examined the molecular mechanisms whereby WT161 enhances bortezomib-induced cytotoxicity. As shown in Supplementary Figure 6A, bortezomib induces modest cleavage of caspase/PARP which is enhanced by WT161, suggesting that combination treatment enhances apoptotic cell death. Previous studies have shown that HDAC6 plays a crucial role in aggresome formation, in which ubiquitinated-protein aggregates are processed through autophagy [25]. Moreover, inhibition of proteasomal degradation enhances aggresomal (autophagic) protein degradation, thereby preventing accumulation of unfolded/misfolded proteins [26]. Indeed, bortezomib triggers significant autophagic response in MCF7 cells, evidenced by LC3 staining, which is almost completely blocked by WT161 (Supplementary Figure 6B). These results further suggest that combined bortezomib with WT161 treatment induces unfolded protein responses (UPR) in MCF7 cells. Importantly, endoplasmic reticulum stress sensor proteins (IRE1α and PERK) are downregulated by WT161, but not by SAHA, treatment (Supplementary Figure 7). Since inhibition of the UPR by inhibiting IRE1α enhances cytotoxicity induced by proteasome inhibitors [27], these data suggest another mechanism whereby WT161 enhances bortezomib-induced cytotoxicity in breast cancer cells. Taken together, these results suggest that single agent anti-tumor activity of WT161 is HDAC6-independent. Importantly, WT161 with bortezomib overcomes bortezomib resistance in breast cancer cells; and HDAC6 inhibition by WT161 can enhance bortezomib-induced cytotoxicity by inhibiting both aggresome/autophagy pathway and UPR in breast cancer cells.

### WT161 analog MAZ1793, which lacks HDAC inhibitory activity, induces significant growth inhibition

**Figure 7:**
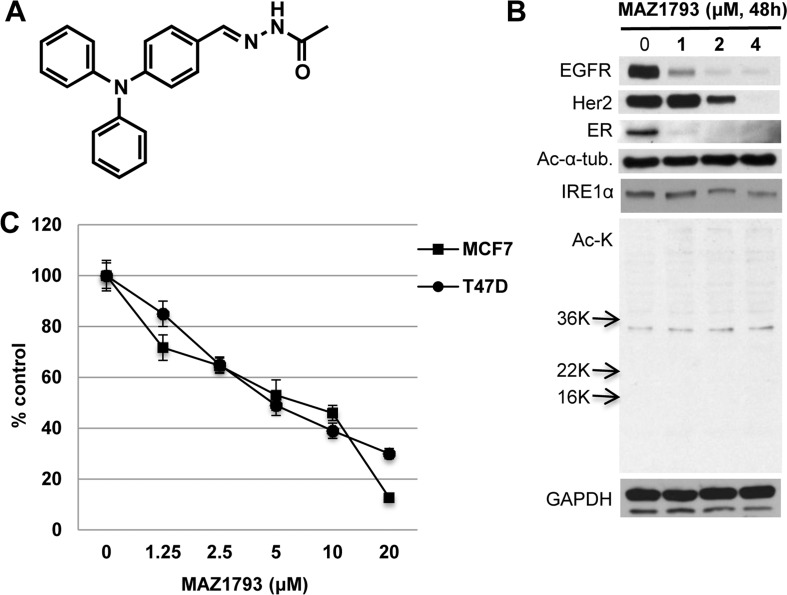
MAZ1793 induces growth inhibition in breast cancer cells **A.** Chemical structure of MAZ1793 is demonstrated. **B.** MCF cells were cultured with MAZ1793 (1 - 4 μM) for 48h. Whole cell lysates were subjected to Immunoblotting using indicated Abs. **C.** MCF7 and T47D cells were cultured with MAZ1793 for 72h. Cell growth was assessed by MTT assay and compared with control.

To precisely define the structure-function relationship between WT161 and the downregulation of growth factors in breast cancer, we prepared a synthetic WT161 analogue MAZ1793, which lacks the zinc-chelating hydroxamate required for HDAC inhibitory activity (Figure 7A). Similar to WT161, MAZ1793 has significant growth inhibitory effect in MCF7 and T47D cells (Figure 7B). Importantly, this growth inhibition is associated with downregulation of EGFR, Her2, ERα and IRE1α; however, no upregulated acetylation of histones or ac-α-tubulin was recognized. These results further confirm that both WT161- and MAZ1793-induced cytotoxicity in breast cancer cells is independent of HDAC inhibition. WT161, but nor MAZ 1793, inhibited bortezomib-induced upregulation of LC3-II, as assessed by immunoblotting which is more quantitative than immunocytochemistry (Supplementary Figure 8).

## DISCUSSION

Chemotherapeutic agents including anthracyclines and taxanes have been combined with targeted therapeutics such as trastuzumab and tamoxifen to improve outcome in subsets of breast cancers. HDAC inhibitors SAHA (vorinostat) [28, 29] and LBH589 (panobinostat) [30], which block class I/II HDACs associated with growth arrest and/or apoptosis, are under evaluation in clinical trials in cancer. HDAC6, a class IIB deacetylase, is a multifunctional, cytosolic protein deacetylase which primarily targets α-tubulin [31]. It plays a key role in aggresomal protein degradation, in which aggregated proteins are processed through autophagy [25]. Recent studies have examined the biologic role of HDAC6 in cancer cells, including breast cancer [32, 33]. However, many HDAC inhibitors block class I and class II HDACs, with only weak inhibitory effect against class IIB HDAC including HDAC6. In this study, we used HDAC6 knockdown and the novel potent and highly selective HDAC6 inhibitor WT161 to characterize the effect of HDAC6 inhibition in breast cancer cells.

We first examined the effect of WT161 on proliferation of breast cancer cells. Although growth of all cell lines was inhibited by WT161 treatment, BT474 (triple-positive) and MDA-MB231 (triple-negative) cells are less sensitive than T47D and MCF7 cells. Importantly, the growth inhibitory effect of WT161 is more potent than other pan-HDAC inhibitors including SAHA and LBH589. This cytotoxicity was associated with caspase/PARP cleavage and is completely blocked by Z-VAD-FMK, suggesting that WT-161 triggers apoptotic cell death. We also observed that WT161 downregulates IAP family member protein XIAP, which protects cells against apoptosis [34, 35]. Specifically, we observed significant cleavage of caspase-7 and PARP triggered by WT161 treatment, consistent with a previous study showing that XIAP primarily blocks caspase-7 [36]. Moreover, transcriptional regulation of XIAP by NF-κB varies depending on cell type [18, 37]; in our study, no NF-κB inhibition by WT161 is recognized, suggesting that WT161-triggered XIAP downregulation occurs independently of NF-κB activity. Importantly, we determined whether WT161-induced cell growth inhibition is due to specific HDAC6 blockade using HDAC6 siRNA knockdown. Surprisingly, no growth inhibitory effect is observed in HDAC6 knockdown cells, indicating that HDAC6 inhibition does not account for WT161-induced cytotoxicity.

Signaling cascades triggered by growth factors/hormones and their receptors mediate growth and progression of breast cancer cells. Since MDA-MB231 breast cancer cell line is triple-negative and relatively resistant to WT161 treatment, we determined whether WT161 alters expression of growth factor/hormone receptors. Indeed, WT161 markedly downregulates EGFR, HER2, and ERα expression. Previous studies have shown that HDAC6 knockdown inhibits EGFR expression in A549 lung cancer cell line, without affecting their growth [19]. Importantly, HDAC6 is associated with the endosomal compartments and controls EGFR trafficking and degradation [20]. Here we observed downregulation of EGFR induced by WT161, but not by other HDAC inhibitors TSA and SAHA that also inhibit HDAC6. Moreover, we did not observe downregulation of EGFR in HDAC6 knockdown cells, indicating that WT161-triggered EGFR downregulation is independent of HDAC6 inhibition. Since WT161 triggers caspase-dependent apoptosis and prior reports show that decreased EGFR may be mediated by activation of caspases [38], we also assessed the role of caspases in WT161-triggered modulation of receptor expression. Our results indicate that EGFR is not a substrate of caspases, since Z-VAD-FMK completely abrogates caspase-7 and PARP cleavage, but does not block WT161-induced EGFR downregulation.

Previous studies have shown that HDAC inhibitors downregulate ERα expression in different models [39, 40]. In our study, ERα downregulation is the most potent effect of WT161 treatment, evidenced both *in vitro* and *in vivo* in a mouse MCF7 xenograft model. Similar to EGFR expression, we did not observe downregulation of ERα in HDAC6-knockdown cells. Our results are in contrast to previous studies showing that HDAC6 deacetylates Hsp90 and regulates its molecular chaperone function [21, 41]. Since EGFR, HER2, and ERα are known client proteins of Hsp90, it is possible that inhibition of HDAC6 downregulates expression of these receptors *via* Hsp90 inhibition. However, in our study hyperacetylation of Hsp90 and downregulation of p-Akt, hallmarks of Hsp90 inhibition, are not induced by WT161 treatment. Our results therefore suggest, in contrast to other HDAC inhibitors, that WT161-triggered ERα downregulation is independent of inhibition of HDAC6 or Hsp90.

The impact of downregulation of EGFR and ERα on cell growth was next studied using small molecule inhibitors, erlotinib and tamoxifen, respectively. Erlotinib did not inhibit growth; however, MCF7 and T47D cells, which are sensitive to WT161, were also sensitive to tamoxifen treatment. Similar results were observed with fulvestrant treatment. These results suggest that downregulation of either EGFR or ERα alone cannot solely account for WT161-induced growth inhibition in breast cancer cells. Since we observed downregulation of EGFR, HER2, and ERα induced by Hsp90 inhibitor 17AAG, we also examined whether 17AAG enhanced downregulation of these receptors is triggered by WT161. The combination of WT161 with 17AAG markedly decreases receptor expression associated with enhanced cytotoxicity, providing the rationale to combine WT161 with Hsp90 inhibitors.

The aggresomal protein degradation pathway is an alternative to the proteasome cascade for degradation of polyubiquitinated misfolded/unfolded proteins [42]. Importantly, HDAC6 has an essential role in aggresome formation, since it can bind both polyubiquitinated proteins and dynein motors, thereby acting to recruit protein cargo to dynein motors for transport to aggresomes [25]. We have previously shown that HDAC6 inhibitor tubacin enhances bortezomib-induced apoptosis in MM cells, without toxicity in normal cells [3, 4, 14]. Others have also shown enhanced cytotoxicity of proteasome inhibitors in combination with HDAC inhibitors in multiple cancer types in both preclinical and clinical studies [43–47]. For example, non selective-HDAC inhibitor vorinostat potentiates cytotoxicity of carfilzomib in human diffuse large B-cell lymphoma cells both *in vitro* and *in vivo* [48]. To date, HDAC inhibitors utilized in prior studies, including borinostat and panobinostat, are broad class I/II HDAC inhibitors; therefore, whether HDAC6 inhibition solely accounts for enhancing cytotoxicity of proteasome inhibitors remains unclear. In this study, we therefore examined whether WT161, a more potent and selective HDAC6 inhibitor, enhances bortezomib-induced cytotoxicity in breast cancer cells. We found that breast cancer cells are more resistant to bortezomib than MM cells. Importantly, WT161 synergistically augments bortezomib-induced breast cancer cytotoxicity. In MM, we have shown that knockdown or blockade of Hsp27 restores sensitivity to bortezomib treatment [49]; conversely, Hsp27 overexpression inhibits doxorubicin-induced apoptosis in breast cancer cells [50]. Moreover, Hsp27 is required for sex steroid receptor (ie, ERα) localization and functioning at the plasma membrane [51]. In preliminary studies, we observed that Hsp27 is highly expressed in MCF7 cells; however, Hsp27 protein expression was not downregulated by WT161 treatment. Ongoing studies are examining whether phosphorylation and acetylation of Hsp27 is modulated by WT161 treatment.

We demonstrated that WT161, in contrast to HDAC inhibitors vorinostat and panobinostat, blocks bortezomib-induced LC3 expression. These results suggest that proteasome inhibition with bortezomib in breast cancer cells induces compensatory aggresomal protein degradation which can be blocked by WT161 *via* HDAC6 inhibition, thereby triggering cell stress followed by apoptotic cell death. Since previous studies have also shown that chronic bortezomib exposure causes a reduction of ERα associated with decreased ERα mRNA level in breast cancer cell lines [52], we also examined whether bortezomib could enhance WT161-induced ERα downregulation. No enhanced effect of combination therapy on ERα expression was observed.

These studies have defined a novel anti-cancer mechanism of a new class of small-molecule chemical probes. WT161 and its chemical derivative MAZ1793 induce a decetylase-independent anti-proliferative effect on breast cancer cells, associated with downregulation of EGFR, Her2, and ERα. Based on the compelling activity observed in these model studies, we have initiated research directed at the identification of the discrete biochemical target of these agents, which may account for the observed selective destabilization of these critical growth regulatory proteins in breast and ovarian cancer. Furthermore, the observed downregulation of EGFR suggests potential therapeutic application more broadly in cancer, including adenocarcinoma of the lung and glioblastoma multiforme. Our data therefore establish a compelling rationale to develop this class of agents for human clinical investigation, and further mechanistic and translational studies in additional tumor models are ongoing.

## MATERIALS AND METHODS

### Cells

MCF7, T47D, MDA-MB231, and BT474 human breast cancer cell lines, as well as RPMI8229 human MM cell lines, were obtained from American Type Culture Collection (Manassas, VA). The cells were cultured in DMEM (Mediatech Inc., Manassas, VA) or RPMI1640 (Mediatech Inc., Manassas, VA) supplemented with FBS (10%), penicillin, streptomycin, and glutamine (Invitrogen, Auckland, New Zealand).

### Reagents

WT161 (C27H30N4O3, MW = 458.55, Supplementary Figure 1) was synthesized by Dr. Bradner's laboratory. MAZ1793 (Figure 7) was generated by Dr. Mazitschek's laboratory. MS275 (entinostat), suberoylanilide hydroxamic acid (SAHA, vorinostat), LBH589 (panobinostat), CUDC-101, tamoxifen, fulvestrant and bortezomib were purchased from Selleck Chemicals (Houston, TX). Erlotinib was purified from discarded patient tablets. MG132 and trichostatin A (TSA) were obtained from Sigma (St. Louis, MO). Z-VAD-FMK and 17-demethoxygeldanamycin (*17-AAG*) were purchased from EMD Chemicals (San Diego, CA). Epidermal growth factor was obtained from R&D Systems (Minneapolis, MN).

### Cytotoxicity assay

Cell growth was assessed by measuring 3-(4,5-dimethylthiazol-2-yl)-2,5-diphenyl tetrazolium bromide (MTT) dye absorbance, as described previously [53]. Briefly, breast cancer cells were harvested using 0.05% trypsin-EDTA (Invitrogen) and distributed into 96-well plates (10,000 - 20,000 cells/well) 24h prior to treatment. All experiments were performed three times in quadruplicate. The experiments were carried out three times, and the average ± SD are demonstrated in Figures.

### Immunoblotting

Cells cultured with WT161 and/or HDAC inhibitors were harvested; washed; and lysed using lysis buffer: 50 mM Tris-HCl (pH 7.4), 150 mM NaCl, 1% NP-40, 5 mM EDTA, 5 mM NaF, 1 mM Na3VO4, 1 mM PMSF, 5 μg/ml leupeptine, and 5 μg/ml aprotinin. Whole cell lysates were subjected to SDS-PAGE, transferred to nitrocellulose membrane (Bio-Rad Laboratories, Hercules, CA), and immunoblotted with specific Abs, as previously described [53]. Antibodies used were anti-acetylated (ac)-lysine (polyclonal), -ac-lysine (K9), -PARP, -caspase-7, -caspase-8, -caspase-9, -IRE1α, -PERK, -XIAP, survivin, cIAP2, -α-tubulin, -GAPDH, -phospho (Tyr1068)-EGF receptor (EGFR), -EGFR, -HER2, -ERα, -phospho (Ser473)-Akt, -Akt, -phospho (Thr202/Tyr204)-ERK, -ERK, -LC3 Abs (Cell Signaling Technology, Danvers, MA). Anti-HDAC6 Ab was purchased from Sigma and Santa Cruz Technology (Santa Cruz, CA), respectively. Anti-ac-tubulin monoclonal Ab was obtained from Sigma-Aldrich (St. Louis, MO). The experiments were carried out at least three times, and representative results are demonstrated in Figures.

### Electrophoretic mobility shift analysis (EMSA)

EMSA was carried out for detection of NF-κB activity, as previously described [54]. Briefly, nuclear extracts from MCF7 cells were obtained using “Nuclear Extraction Kit^®^” (Panomics, Fremont, CA). Double-stranded NF-κB consensus oligonucleotide probes (Promega, Madison, WI) were end labeled with [γ^32^P]ATP (10 mCi/ml, PerkinElmer, Boston, MA). Binding reactions containing 0.035 pmol/μl of oligonucleotide and 8 μg of nuclear protein were conducted at room temperature for 30 min in binding buffer (10 mM Tris-HCl, pH 7.5, 50 mM NaCl, 1 mM MgCl_2_, 0.5 mM EDTA, 0.5 mM DTT, 4% glycerol (v/v), and 0.5 μg poly (dI-dC) (Pharmacia, Peapack, NJ). The samples were loaded onto a 4% polyacrylamide gel, transferred to Whatman paper, and visualized by autoradiography. The experiments were carried out at least three times, and representative results are demonstrated in Figures.

### RT-PCR

Gene expression of EGFR, HER2, and ERα was examined by RT-PCR and normalized to expression of GAPDH. Briefly, total RNA was extracted from untreated or bortezomib-treated RPMI8226 cells using RNeasy Kit and RNase-Free DNase Set (Qiagen, Valencia, CA). cDNA was synthesized using first strand cDNA synthesis Kit (SuperArray Bioscience, Frederick, MD). Gene-specific oligonucleotide primers (Invitrogen, Carlsbad, CA) included: EGFR, forward 5′-AGGCACGAGTAACAAGCTCAC-3′ and reverse 5′-ATGAGGACATAACCAGCCACC-3′; ERα, forward 5′-CCCACTCAACAGCGTGTCTC-3′ and reverse 5′- CGTCGATTATCTGAATTTGGCCT-3′; HER2, forward 5′- TGACACCTAGCGGAGCGAT-3′ and reverse 5′- GGGGGATGTGTTTTCCCTCAA-3′; as well as GAPDH, forward 5′-AATCCCATCACCATCTTCCA-3′ and reverse 5′-TGGACTCCACGACGTACTCA-3′. The experiments were carried three times, and representative results are demonstrated in Figures.

### Transient transfection of HDAC6 siRNA

MCF7 cells were transiently transfected with HDAC6 siRNA (ON-TARGETplus^®^ human HDAC6, Dharmacon Inc., Lafayette, CO) using “Cell Line Nucleofecto™ Kit V,” according to manufacturer's (Amaxa Biosystems, Gaithersburg, MD) instructions. Following transfection, MCF7 cells were subjected to Western blotting and MTT assay, in the presence or absence of bortezomib. The experiments were carried out at least three times, and representative results are demonstrated in Figures.

### MCF-7 xenograft murine model

Female NCr *nu/nu* mice (Charles River Laboratories, Wilmington, MA) were subcutaneously (sc) implanted with 17ß-estradiol-sustained release pellets (0.18 mg, 60 day release time, Innovative Research, Sarasota, FL). Mice were inoculated sc with 5 × 10^6^ MCF-7 cells suspended in 30% Matrigel (BD Biosciences, San Diego, CA). After 1 month of growth, mice with measurable tumors (> 50 mm^3^) were assigned into cohorts receiving intraperitoneal WT161 daily (80 mg/kg) or into a control group receiving vehicle alone (10% DMSO, 90% PBS). Caliper measurements of the longest perpendicular tumor diameters were performed every alternate day to estimate the tumor volume, using the formula volume = ½ x L x W^2^. Animals were sacrificed when tumors reached 2 cm or if the mice appeared moribund. For each animal, relative tumor volume was determined by normalizing data to the baseline tumor volume for that animal at the start of treatment. Statistical significance was determined by 2-way ANOVA analysis. All animal studies were performed under the auspices of IACUC approved protocols.

### Immunohistochemistry

Immunohistochemistry (IHC) was performed as described previously [53]. The primary Abs (anti-ER, -EGFR, -HER2, -ac-tubulin, -TUNEL) were visualized with the corresponding biotinylated Ab coupled to streptavidin-peroxidase complex (Vector Laboratories, Burlingame, CA). All Abs, conditions, and reactivities were tested in positive control slides. Histological photo micrographs were taken using a Leica DM200 microscope (aperture HC PLANs 10X/22, objective lenses: N PLAN 100X/1.25 oil), and a SPOT Insight QE Model camera with SPOT Advanced acquisition software (Diagnostic Instruments, Sterling Heights, MI).

### Immunocytochemistry

Immunocytochemistry (ICC) was carried out as described previously [4]. Briefly, MCF7 cells were treated with WT161 (1 μM) in the presence (20 nM) or absence of bortezomib for 24h. Cells were then fixed in cold absolute acetone and methanol for 10 minutes. After fixation, cells were washed in PBS and then blocked for 60 minutes with 5% fetal bovine serum in PBS. Slides were then incubated with anti-LC3 antibody (Cell Signaling) at 4°C for 24 hours, washed in PBS, incubated with goat anti-mouse IgG-fluorescein isothiocyanate for 1 hour at 4°C, and analyzed using Nikon E800 fluorescence microscopy. Images were taken with objective lenses (N Plan 60x/1.25 oil), using a SPOT Insight QE model camera with SPOT Advanced acquisition software (Diagnostic Instruments Inc, Sterling Heights, MI). The experiments were carried out at least two times, and representative results are demonstrated in Figures.

### Statistical analysis

Statistical significance of differences observed in drug-treated *versus* control cultures was determined using the Wilcoxon signed-ranks test. The minimal level of significance was *p* < 0.05. The interaction between HDAC inhibitors and bortezomib was analyzed by isobologram analysis using the CalcuSyn software program (Biosoft, Ferguson, MO) to determine whether the combination was additive or synergistic; a combination index (CI) < 1.0 indicates a synergistic cell growth inhibitory effect.

## SUPPLEMENTARY MATERIALS FIGURES


