# Correction: HDAC6 inhibitor WT161 downregulates growth factor receptors in breast cancer

**DOI:** 10.18632/oncotarget.28051

**Published:** 2021-08-17

**Authors:** Teru Hideshima, Ralph Mazitschek, Jun Qi, Naoya Mimura, Jen-Chieh Tseng, Andrew L. Kung, James E. Bradner, Kenneth C. Anderson

**Affiliations:** ^1^ Department of Medical Oncology, Dana-Farber Cancer Institute and Harvard Medical School, Boston, MA, USA; ^2^ Center for Systems Biology, Massachusetts General Hospital, Harvard Medical School, Boston, MA, USA; ^3^ Lurie Family Imaging Center, Dana-Farber Cancer Institute, Boston, MA, USA; ^4^ Department of Pediatric Oncology, Dana-Farber Cancer Institute and Children’s Hospital Boston, Boston, MA, USA; ^5^ Department of Hematology, Chiba University Hospital, Chiba, Japan; ^6^ PerkinElmer Inc., Hopkinton, MA, USA; ^7^ Memorial Sloan Kettering Cancer Center, New York, NY, USA; ^8^ Novartis Institutes for BioMedical Research, Cambridge, MA, USA

**This article has been corrected:** The CONFLICTS OF INTERESTS section has been updated as shown below:

## CONFLICTS OF INTEREST

K.C. Anderson is on the advisory board of Celgene, Gilead, Millennium and Bristol Myers Squibb; and is a scientific founder with financial interest in Acetylon, Oncopep and C4 Therapeutics. JB is a scientific founder with financial interest in Acetylon.

Original article: Oncotarget. 2017; 8:80109–80123. 80109-80123. https://doi.org/10.18632/oncotarget.19019

