# Supplementary material for: HDAC6 inhibitor WT161 downregulates growth factor receptors in breast cancer
Source: Oncotarget. 2017 Jul 5;8(46):80109–23. doi: 10.18632/oncotarget.19019 (PMC5655183; doi:10.18632/oncotarget.19019)
Supplement: Supplementary file 1 [file oncotarget-08-80109-s001.pdf]

HDAC6 inhibitor WT161 downregulates growth factor receptors in breast cancer

Supplementary Information

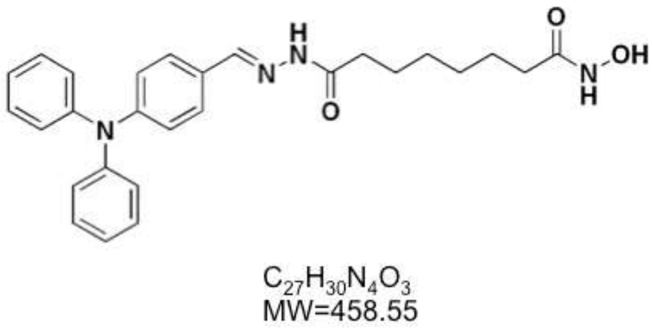

Supplementary Figure 1 : Chemical structure of WT161.

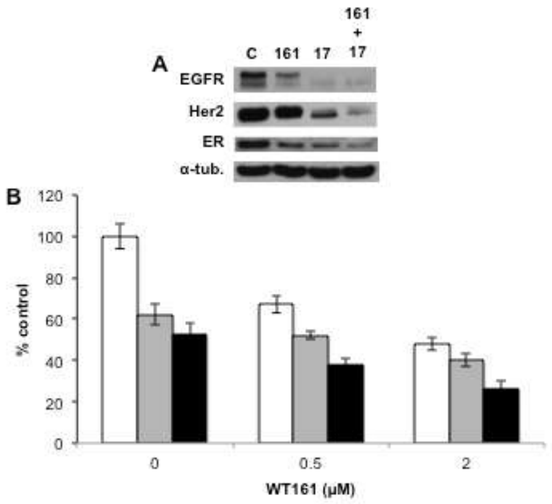

Supplementary Figure 2. WT161 enhances 17AAG-induced cytotoxicity.

(A) MCF7 cells were cultured with WT161 (0.5 μM) and/or 17AAG (0.25 μM) for 24h. Whole cell lysates were subjected to immunoblotting with indicated Abs. (B) MCF7 cells were cultured for 48h with WT161 (0.5 and 2 μM) in the presence of DMSO control (□), and 125 nM (■) or 500 nM (■) 17AAG. Cell growth was assessed by MTT assay.

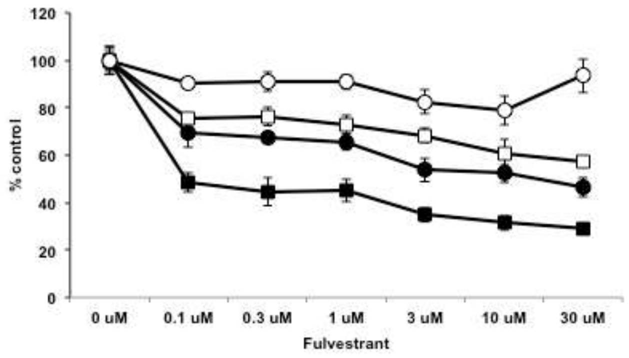

Supplementary Figure 3. Fulvestrant inhibits breast cancer cell growth.

MCF7 (●), T47D (■), BT474 (□), and MDA-MB231 (○) cells were cultured with fulvestrant (0 - 30 µM) for 72h. Cell growth was assessed by MTT assay.

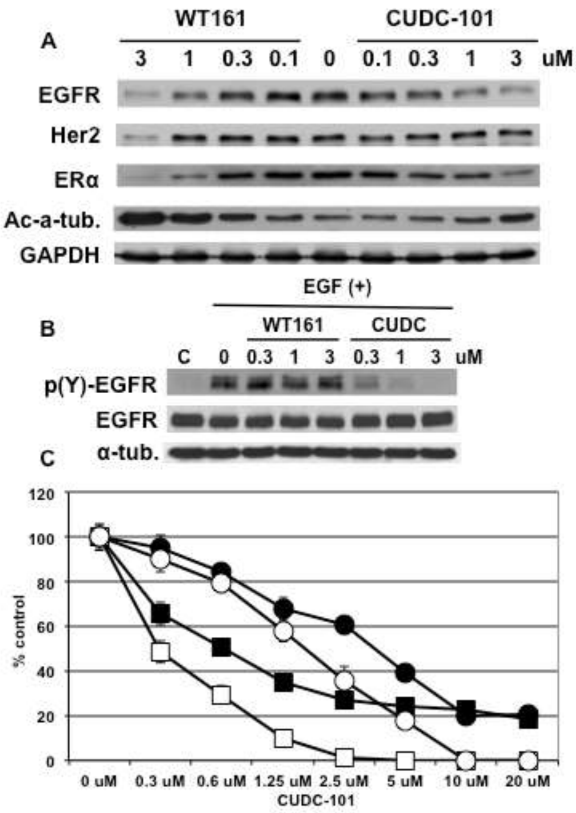

Supplementary Figure 4. WT161 does not inhibit EGFR phosphorylation.

(A) MCF7 cells were cultured with WT161 (0.1 - 3 µM) or CUDC-101 (0.1 - 3 µM) for 24h. (B) MCF7 cells were cultured with WT161 (0.3 - 3 µM) or CUDC-101 (0.3 - 3 µM) for 4h. The cells were then stimulated with EGF (50 ng/ml) for 10 min. In each case, whole cell lysates were subjected to immunblotting with indicated Abs. (C) MCF7 (●), T47D (■), BT474 (□), and MDA-MB231 (○) cells were cultured with fulvestrant (0 - 20 µM) for 72h. Cell growth was assessed by MTT assay.

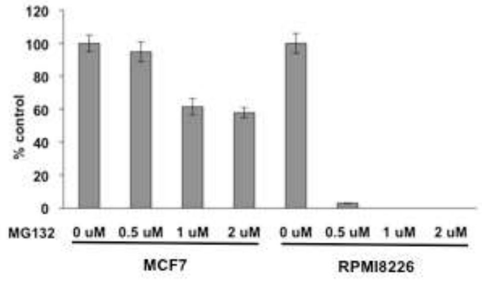

Supplementary Figure 5. MCF7 cells are resistant to MG132.

MCF7 and RPMI8226 cells were cultured with MG132 (0 - 2 µM) for 48h. Cell growth was assessed by MTT assay.

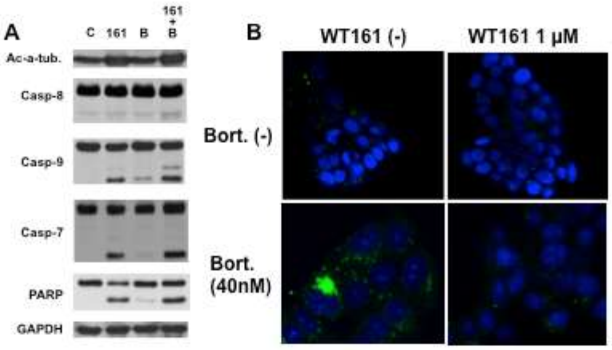

Supplementary Figure 6. WT161 blocks bortezomib-induced autophagy.

(A) WT161 enhances bortezomib-induced caspase/PARP cleavage and blocks autophagy. MCF7 cells were cultured with WT161 (2 μM) for 24h, in the absence or presence of bortezomib (20 nM). Whole cell lysates were subjected to immunoblotting with indicated Abs. (B) MCF7 cells were treated with 1μM WT161 for 24h, in the presence of DMSO control or bortezomib (40 nM). Green indicates bortezomib-induced LC3 accumulation, and blue indicates nuclear DAPI staining, respectively, evidenced by fluorescence microscopic analysis.

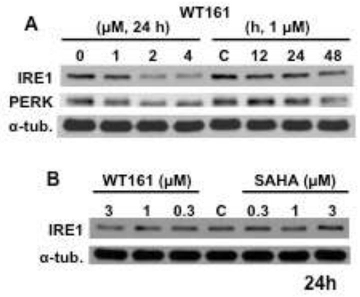

Supplementary Figure 7. WT161 downregulates IRE1α.

(A) MCF7 cells were cultured with WT161 (1 - 4 μM) for 24h or 1 μM WT161 for for 12 - 48h. (B) MCF7 cells were cultured with WT161 (0.1 - 3 μM) or SAHA (0.1 - 3 μM) for 24h. Whole cell lysates were subjected to immunoblotting with indicated Abs.

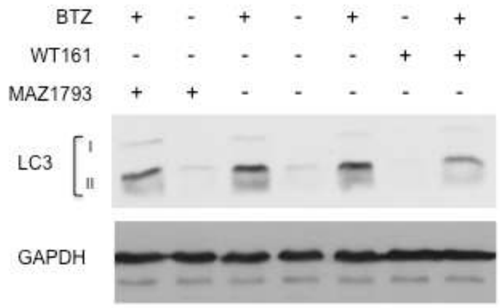

Supplementary Figure 8. MAZ1793 does not block bortezomib-induced upregulation of LC3-II.

MCF7 cells were treated with 1µM WT161 or 2µM MAZ1793 for 24h, in the presence of DMSO control or bortezomib (BTZ, 40 nM). Whole cell lysates were subjected to immunoblotting with indicated Abs.
